# The *Mammalian Adult Neurogenesis Gene Ontology* (MANGO) Provides a Structural Framework for Published Information on Genes Regulating Adult Hippocampal Neurogenesis

**DOI:** 10.1371/journal.pone.0048527

**Published:** 2012-11-05

**Authors:** Rupert W. Overall, Maciej Paszkowski-Rogacz, Gerd Kempermann

**Affiliations:** 1 CRTD – Center for Regenerative Therapies Dresden, Technische Universität Dresden, Dresden, Germany; 2 UCC – University Cancer Center, Medical Faculty, Technische Universität Dresden, Dresden, Germany; 3 DZNE, German Center for Neurodegenerative Diseases, Dresden, Germany; IRB Barcelona, Parc Cientific de Barcelona and CIBERNED (ISCIII), University of Barcelona, Spain

## Abstract

**Background:**

Adult hippocampal neurogenesis is not a single phenotype, but consists of a number of sub-processes, each of which is under complex genetic control. Interpretation of gene expression studies using existing resources often does not lead to results that address the interrelatedness of these processes. Formal structure, such as provided by ontologies, is essential in any field for comprehensive interpretation of existing knowledge but, until now, such a structure has been lacking for adult neurogenesis.

**Methodology/Principal Findings:**

We have created a resource with three components 1. A structured ontology describing the key stages in the development of adult hippocampal neural stem cells into functional granule cell neurons. 2. A comprehensive survey of the literature to annotate the results of all published reports on gene function in adult hippocampal neurogenesis (257 manuscripts covering 228 genes) to the appropriate terms in our ontology. 3. An easy-to-use searchable interface to the resulting database made freely available online. The manuscript presents an overview of the database highlighting global trends such as the current bias towards research on early proliferative stages, and an example gene set enrichment analysis. A limitation of the resource is the current scope of the literature which, however, is growing by around 100 publications per year. With the ontology and database in place, new findings can be rapidly annotated and regular updates of the database will be made publicly available.

**Conclusions/Significance:**

The resource we present allows relevant interpretation of gene expression screens in terms of defined stages of postnatal neuronal development. Annotation of genes by hand from the adult neurogenesis literature ensures the data are directly applicable to the system under study. We believe this approach could also serve as an example to other fields in a ‘bottom-up’ community effort complementing the already successful ‘top-down’ approach of the Gene Ontology.

## Introduction

Adult hippocampal neurogenesis is a complex multi-stage process. An ever increasing number of publications deal with the role of single genes in the control and regulation of adult neurogenesis. A crucial question becomes how this information can be integrated to form coherent concepts about the molecular bases of adult neurogenesis [1]. As they stand, the data fail to provide a comprehensive picture because they often either use different nomenclatures or the same nomenclature with a different understanding. Similar problems across biology have led to initiatives to develop defined standardized vocabularies (i.e. ontologies) that allow the integration of large amounts of data across studies and fields of research [1]–[3]. In the end, useful ontologies are community efforts that need to integrate with existing related initiatives and respect a growing set of rules that evolve to accomplish such tasks [4].

The best known initiative of this type is the Gene Ontology (GO) [5]–[7]. GO provides a “controlled vocabulary to describe gene and gene product attributes in any organism” (www.geneontology.org). The immense scope of the GO project means that very specialist terms, particularly those only applicable to a subset of organisms, may not be present in the ontologies. This is currently the case for adult hippocampal neurogenesis. The GO effort is by no means static, however, and is continuously being extended with new terms, so that children of the term “neurogenesis” (GO:0022008) now reach as far as focused terms like “regulation of glial cell proliferation” (GO:0060253) and “regulation of neuron differentiation” (GO:0045664). We believe that specialist categories could also be provided as community-driven extensions of the GO initiative, and we present here a step in this direction with the *Mammalian Adult Neurogenesis Gene Ontology* (MANGO).

The definitions in the present study make reference to structural principles of GO, so that a link between our evolving adult neurogenesis ontology and GO will be possible. The ultimate goal of MANGO is to allow the annotation of genes with regard to their relevance in adult neurogenesis and to facilitate the building of integrative models that describe the molecular mechanisms underlying adult neurogenesis. Unfortunately, this cannot be achieved by a straight application of the building principles of GO alone because GO does not yet capture cell types and their development, i.e. the transition between cell types, a feature central to adult neurogenesis.

Our hope is that MANGO should be a common goal for the whole adult neurogenesis field and the current study does not intend to foreclose–rather initiate interest in–just such a community effort. It is clear that, to gain full impact, MANGO will require further formalization than presented here, and that gene annotation is an ongoing process–but the resource we present is already immensely useful, and we describe several examples of how it can be already applied to problems in adult neurogenesis research.

## Results

### Development of the Core Ontology

**Figure 1:**
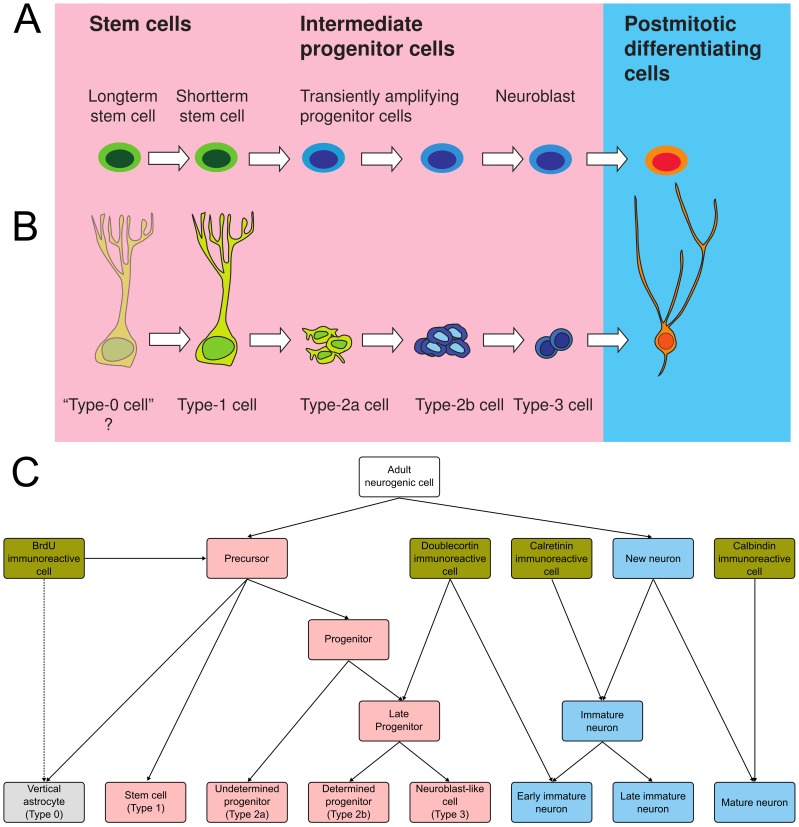
Development of the ontology. **A**. The descriptive nomenclature used to describe adult hippocampal neurogenesis has been aligned with the basic models of stem cell biology as well as the stages during cortical development. **B**. Stages in the process of adult hippocampal neurogenesis following the widely adopted scheme from Kempermann *et al.* [8]. **C**. A graphical overview of the core ontology. Relationships between terms are represented as a tree, so that the nesting of child terms is easily seen. Each of the lowest level ‘leaf’ terms corresponds to a cell type according to the scheme above.

Adult hippocampal neurogenesis is a process involving the transformation of a proliferating stem cell into a functionally mature dentate gyrus granule cell. We thus decided to base our ontology on a hierarchical organization of ‘cell types’–more correctly stages–comprising this maturation process (**Fig. 1A**). The most widely used description of identifiable stages in the course of adult hippocampal neurogenesis was proposed by us previously [8] and this model was used as the backbone of the new ontology (**Fig. 1B**). Although variations on this model have been proposed [9]–[11] and despite an increase in the number of markers described in the literature [8]–[10], [12]–[17], the definition of the different cell types or stages of development has not been significantly sharpened. Therefore, because of its wide acceptance and in the absence of a sufficiently different and better model, we have used our original schema [8] as the basis for the descriptions of ‘cell types’ as detailed in Methods. Following these descriptions, formal definitions were given to each term such that daughter terms in the final ontology are related by ‘is_a’ relationships to parents. Although the four histological markers, BrdU, DCX, calretinin and calbindin are equivocal regarding the cell types they identify, these are so commonly employed in the literature, we felt they warranted inclusion in the ontology.

The resulting final ontology (**Fig. 1C**) allows the role of genes to be annotated to various levels of the hierarchy–depending on the resolution of the available information. In addition to the core ontology, we also included the cell-type categories ‘astrocyte’, ‘oligodendrocyte’ and ‘granule cell neuron’ which, although they did not fit into the ontology structure, describe important aspects of adult neurogenesis.

### Annotation of Genes from the Literature

As a basis for the complete gene list (**Table S1**), we used a manually curated list that has been maintained over the past years, covering 189 genes. We extended this list by carrying out a search for genes related to “adult neurogenesis” at GoGene. Key publications describing the role of each gene in adult neurogenesis were used as the basis of the database. In addition to this initial survey, an automated search has been established to identify new publications on a daily basis. This ongoing review of the literature, together with the manual submission of manuscripts, provides an up-to-date list of publications for curation. The database, version 2.0 at the time of writing, comprises 257 annotated manuscripts covering 228 genes. Only 58 genes are represented by more than 1 report. Although one gene (*Vegfa*) has attracted 7 studies, clearly genetic information in this field is still very thinly spread.

The curation of each manuscript involved identifying statements pertaining to the activity of a gene or gene product in one of the defined cell types.The matrix of possible activity/process vs. cell type combinations is presented in **Table 1**. Annotation was restricted to normal gene function, so that studies dealing with disease mutations or the role of genes under pathological conditions were not included. Each gene was annotated to the deepest term of the ontology possible, given the information available from the primary publication. Because each higher-level term also includes annotated genes from all of its child terms, data from studies with different design can be integrated and searches can be carried out at different levels of focus. This approach can yield insights not possible from a traditional review of the literature. In addition to the position in the core ontology, genes were also annotated to a process or outcome measure, and gene effect was noted as either positive, negative or neutral.

**Table 1:** The cell type × process matrix.

	Cell types							
	Precursor cells					New Neurons		
		Progenitor cells				Immature neurons		
	‘Stem cell’	DCX cells				CR cells		
	Type-0	Type-1	Type-2a	Type-2b	Type-3	DCX/CR	CR/?	CB
**Process**								
Proliferation	○	•	•	•	•			
Differentiation			•	•	○			
Migration				•	•	○		
Survival				•	•	•	•	○
Dendrite development					•	•	•	○
Axon development					○	•	•	○
Maturation				○	•	•	•	○
**Outcome**								
Active granule cell function						○	○	•
Gene expression	•	•	•	•	•	•	•	•
Cell numbers	•	•	•	•	•	•	•	•

The cell types refer to the definitions in the text and to the underlying model depicted in **Figure 1B**. •, primary association between cell type/stage and process. ○, association possible but either only hypothetical, only indirect, or not confirmed.

Seventy-five of the included studies are on rats, two on monkeys, and one on guinea pigs. All remaining reports are on mice. Because of the overwhelming focus on the mouse in the literature, the Gene ID in the table always refers to the *Mus musculus* gene, in order to facilitate further analysis.

**Figure 2:**
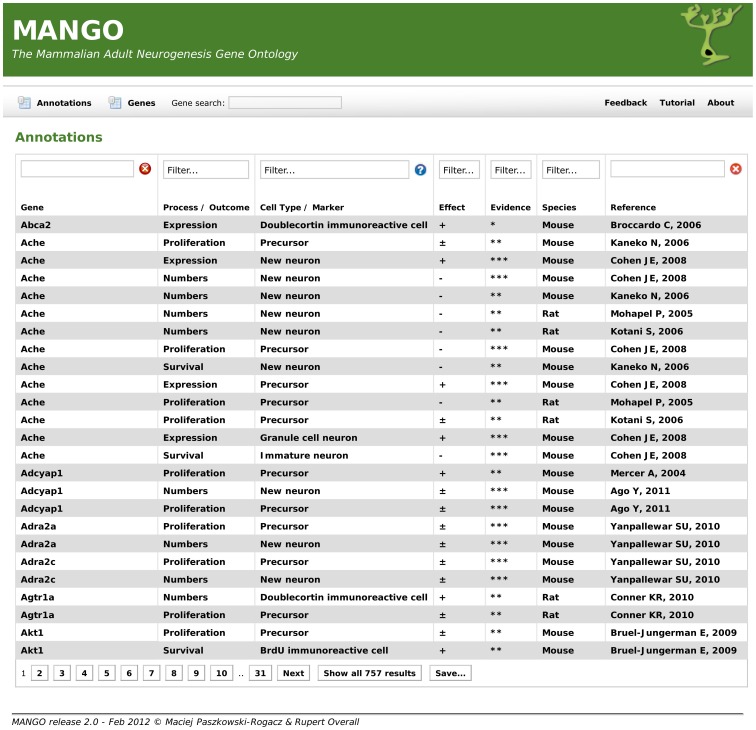
Publicly accessible interface for the MANGO project. MANGO provides a searchable online database, accessible under http://adult-neurogenesis.de/resources/mango, allowing users to search for genes, cell types, processes and other variables. Using the ontology, searches are extended to include child categories, and all results can be downloaded for further analysis. This database will continue to be updated as new genetic information on adult neurogenesis becomes available. The complete information as of publication of this article is also found in **Table S1**.

The resulting complete table is found in the supplemental material (**Table S1**). We have also made the database available in a searchable form at http://www.adult-neurogenesis.de/resources/mango/ (**Fig. 2**).

### An Overview of the Neurogenesis Literature

**Figure 3:**
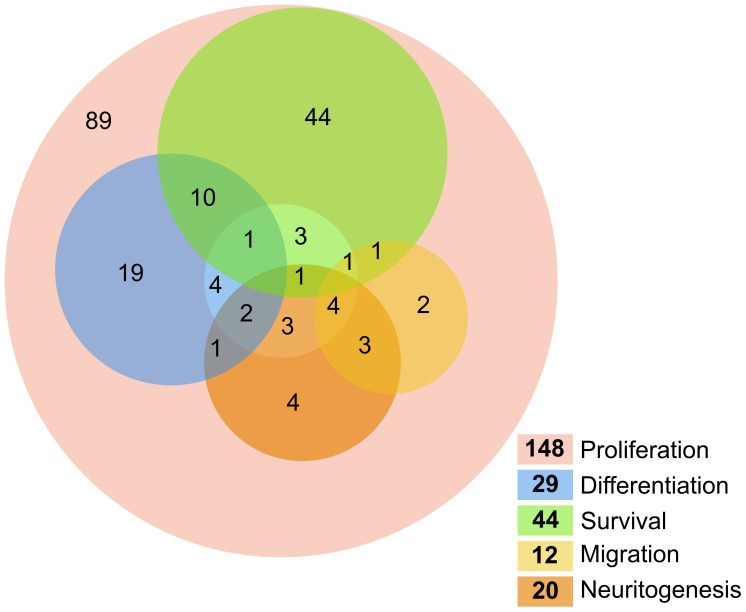
A Venn-type diagram of processes in adult neurogenesis. Gene annotations for the 5 defined processes in the ontology are summarized to show intersections between processes (where a gene is annotated to two or more categories). The ‘proliferation’ set is drawn as a ring (pink) to allow for correct overlap with the other sets. The remaining 4 sets, ‘differentiation’ (blue), ‘survival’ (green), ‘migration’ (yellow), and the composite term ‘neuritogenesis’ (orange; see text for details), are overlaid as semi-transparent circles to highlight the overlaps between multiple terms. Numbers in bold on the color legend are totals for each process category, whereas numbers on the diagram indicate the number of genes annotated to each intersection subset.

To gain an overview of the relationships between processes, genes annotated to the five ‘process’ categories were displayed in a Venn-type diagram in which overlapping categories can be visualized (**Fig. 3**). To maintain readability of the diagram, we collapsed the terms ‘dendritogenesis’, ‘axonogenesis’ and ‘maturation’ into ‘neuritogenesis’, given the low number of publications in these three related categories.

Most studies (165/257) cover ‘proliferation’, the vast majority generically for ‘precursor cells’ in the sense of cells labeled with bromodeoxyuridine. Only 13 studies further break down the analysis to defined precursor cell populations, but these studies reveal that proliferative activity might differ between the different precursor cell stages.

Few studies directly address differentiation in the sense of fate choice and the term is often used rather loosely. There are only 31 studies on ‘differentiation’, only 6 of which address gliogenesis. Eight studies report differentiation of new neurons as unchanged. There is only one report on oligodendrogenic differentiation.

Information on migration is very limited, only 12 genes have been investigated in this context, usually based on DCX as marker.

Survival is an important phenotype, however, showing large overlap with the outcome ‘numbers of new neurons’. If studies differentiate between them, these two measures are usually concordant. A notable exception is *Bax*, where survival is reduced but results in unchanged net neurogenesis, because of an increased level of proliferation.

While 15 studies address dendritogenesis, there is only one single entry on axonogenesis (*Ccdc88a*). Six reports cover ‘maturation’ as measures of neuronal integration beyond the extension of neurites.

Surprisingly, out of the total of 257 published reports on adult neurogenesis, only 102 have net neurogenesis (i.e. ‘numbers’ of ‘new neurons’) as a reported outcome measure. A common, problematic, surrogate marker is the number of DCX expressing cells. Seven studies include effects of adult neurogenesis at the level of total granule cell numbers.

From this overview, it can be immediately seen that information about proliferation is vastly overrepresented, and that very few studies have addressed more than one or two processes.

### Patterns of Regulation and Molecular Pathways

**Figure 4:**
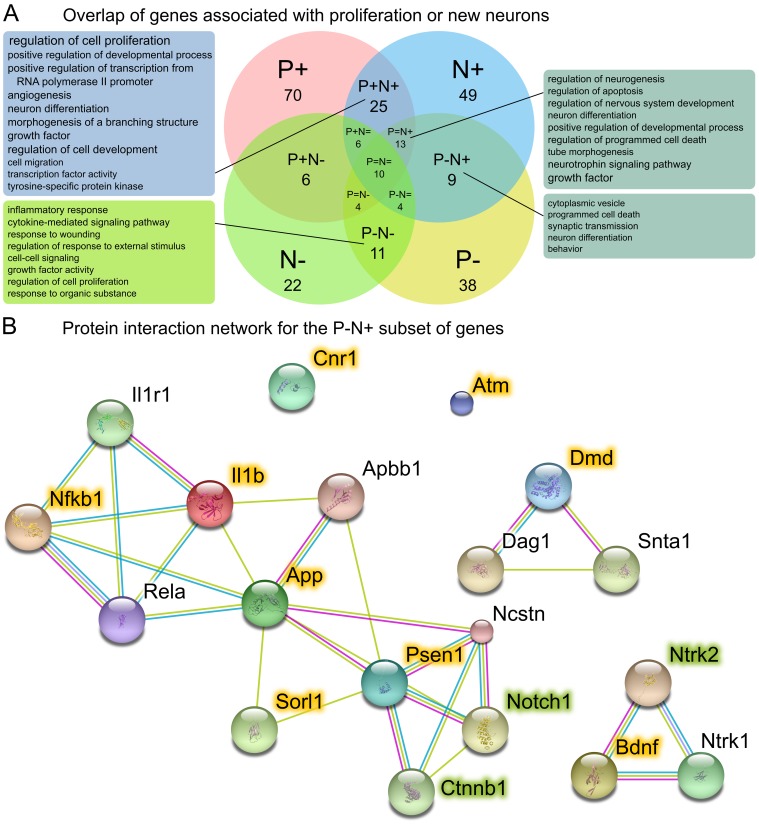
Functional annotation of genes involved in proliferation and/or neurogenesis. Genes annotated as up- (P+) or downregulating (P−) proliferation of precursor cells, or increasing (N+) or decreasing (N−) neurogenesis (numbers of new neurons) are shown as a Venn diagram. Neutral genes (P = and N = ) are depicted as the intersection between the positive and negative sets. Numbers for P+, P−, N+ and N− are total number of genes annotated to these categories. All other numbers refer to the intersection only. Gene function enrichment analysis was carried out for the four largest intersections and enriched annotation terms are shown in the boxes–larger font size indicating more significant enrichment. The 7 genes that were negatively associated with proliferation yet involved in upregulation of neurogenesis (P−N+) were used to query the STRING protein interaction database. The resulting network was extended by including 10 interaction partners from the STRING database to reveal a network linking many of the original members. Network members from the original query (the 7 P−N+ genes) are highlighted in yellow. Genes highlighted in green were not in the P−N+ list but are also present in the MANGO database.

While the compressed literature review of **Figure 3** (and the underlying **Table S1**) provides a useful overview, considering the direction of regulation allows another level of analysis. We focused here on the relationship between precursor cell proliferation, the trait with most available published information, and net neurogenesis (i.e. ‘numbers’ of ‘new neurons’), both of which phenotypes can be either up- or downregulated or remain unchanged. We searched for genes which regulate proliferation (‘Process/Outcome’ = ‘Proliferation’) and those which regulate neurogenesis (‘Process/Outcome’ = ‘Numbers’ and ‘Cell Type/Marker’ = ‘New neuron’) and separated these by whether they are involved in up- or downregulation (‘Effect’ = ‘+’ or ‘−’). The overlap of the resulting gene lists (**Fig. 4A**) shows that proliferation and net neurogenesis are not only, as might be expected, regulated in parallel but there are cases in which the directionality of regulation is uncoupled–such as downregulation of proliferation but upregulation of neurogenesis. We used DAVID, an online functional annotation tool which includes annotations from GO, to identify enriched annotation categories for the intersections. Each intersection could be shown to contain genes involved in distinct functional processes (**Fig. 4A**), further emphasizing the genetic regulation of proliferation and new neuron survival as being distinct processes. We examined the nine genes in this intersection (P−N+) more closely. The P−N+ gene list was submitted to STRING, a protein interactome database, to explore potential links between these nine genes. The result is depicted in **Figure 4B**. When the 10 best matches from the STRING database were added to the interaction network, three new genes were included which were not in the original P−N+ list, but which are already in the MANGO database. The remaining proteins in this extended STRING network (*Apbb1, Dag1 Il1r1, Ncstn*, *Rela* and *Snta1*) can now be investigated with respect to their effects on adult neurogenesis within this newly found context (see discussion).

Similar analyses are not yet feasible for most other combinations of processes or, even more important, broken down to effects at different cell types, but this is merely a result of the low number of annotated genes for which information is yet available and this situation will certainly improve as the database evolves over time.

### Gene Set Enrichment

**Figure 5:**
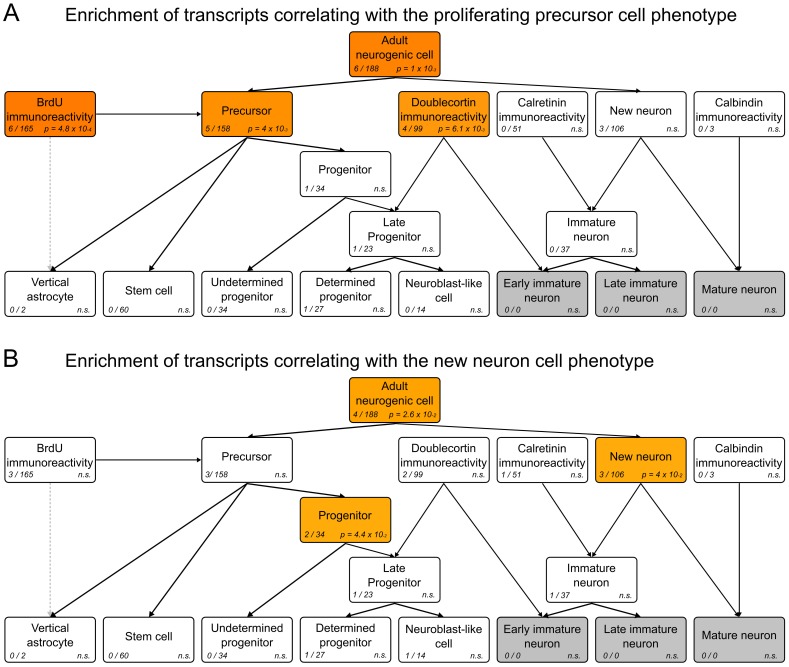
Enrichment analysis of gene lists associated with adult neurogenesis. Transcripts correlating with neural stem cell proliferation (A) or new-born neuron number (B) in a panel of mouse strains were analyzed for enrichment in each of the MANGO categories. The hypergeometric test was used to calculate significance scores in enriched categories. For each term, the number of query genes identified and the total number of genes annotated to the term are shown. Terms are colored darker shades of red corresponding to more significant results. The three terms grayed out do not yet contain gene annotations.

One of the key advantages of having genes annotated to a structured ontology is the ability to investigate gene sets of interest and discover whether they are enriched for certain functional annotations. This is one of the main uses of the Gene Ontology and is also an intended use of MANGO. We have done just such an analysis with two gene lists pertaining to different aspects of neurogenesis. In a previous study, we had identified genes whose expression patterns in the brain correlate to histology phenotypes measured in a collection of recombinant inbred mouse strains [18]. This dataset enables the calculation of correlations between phenotypes and genes which exhibit similar expression profiles across the different strains. We thus searched for genes correlating to either of two phenotypes ‘PROL’ (numbers of Ki67-positive staining cells) or ‘NEUR’ (numbers of BrdU/NeuN double-positive cells 4 weeks after BrdU labeling)–but not to both phenotypes (‘exclusive OR’). We were interested in seeing if members of the resulting two gene lists were more likely to be present in any of the MANGO categories. Using Fisher’s hypergeometric test with Bonferroni correction, we calculated the enrichment of each ontology term for our query gene lists. The results in **Figure 5** and **Table S2** show the genes correlating to the ‘PROL’ phenotype to be highly enriched in categories associated with early proliferative stages, ‘BrdU’ (*P* = 4.8×10^−4^), ‘Precursor’ (*P* = 4×10^−3^) and ‘Doublecortin’ (*P* = 6.1×10^−3^), whereas the genes correlating to ‘NEUR’ were significantly enriched for the terms ‘Progenitor’ (*P* = 4.4×10^−2^) and ‘New neuron’ (*P* = 4×10^−2^). This example confirms that trends in an already characterized microarray dataset can be identified, and hints at the sort of information that will become retrievable from MANGO as the body of published literature in this field grows.

## Discussion

In this study we show that the systematic integration of published information on adult neurogenesis reveals insight not apparent from the individual studies and allows placing these studies into the context of the field as a whole. By enabling the mapping of results on to a standardized ontology framework, previously isolated studies–whose results might have been only compatible with immediately related reports–may now be usefully compared.

With respect to the ‘state of the art’ in the field, we found that the published information on genes in adult neurogenesis is biased in two ways: most studies about the roughly 200 genes covered so far are single reports on single genes, and selection of the genes studied was led by the preference of researchers rather than systematic methods. Although a valid and fruitful approach, this subjectivity creates a bias towards known candidate genes. The genetic information on adult neurogenesis to date is not only relatively limited, thinly spread and overemphasizing ‘usual suspects’, it is also skewed due to rather broad readout parameters, e.g. BrdU-labeled cells, with few and sometimes rather non-specific end points (‘proliferation’). This should not sound overly critical to the colleagues who published these reports. To a certain degree, this picture is immanent in the way biological research is done, but we need to be aware of the consequences on how we perceive the genetic bases of adult neurogenesis.

Statements are retrieved from each manuscript independently of other statements and are not summarized in any way before entry into the database. This means that the same gene may be present with different–even opposite–effects. This is especially the case when comparing published effects between in vivo and in vitro experiments. We do not attempt to resolve such discrepancies, rather aiming to faithfully reflect the literature.

The relation of published information to one common underlying model, the annotation of the reports to a standardized vocabulary, and the analysis across all published genes revealed interesting–sometimes counterintuitive–patterns of regulation at different stages of development. Given the still relatively low number of genes presently in the database, however, and the lack of studies that have analyzed specific cell types in greater detail, this analysis can not yet be extended to the ultimately intended resolution. We have also opted to exclude genes for which only a pathogenic role has been reported. In response to injury or disease, the regulation of adult neurogenesis can become dysregulated so that the terms and their interrelations described in the ontology may no longer hold true. Some pathologies are also associated with altered expression of many genes which in turn affect consistent annotation of these genes to the MANGO terms.

Nevertheless, the current scale of the project is already able to provide useful results, as demonstrated by the discovery of a group of genes associated with downregulation of proliferation but upregulation of new neuron number. Use of the STRING database suggested several candidates that are closely associated with neurogenesis-related phenotypes and may be involved themselves in adult hippocampal neurogenesis. Of these, *Rela*, for example, has been implicated in neurogenesis in the subventricular zone [19] and *Apbb1* is known to be important for neurogenesis during development [20] and in cortical neural progenitor culture [21]. Indeed *Il1r1* [22] and *Ncstn* [23] have been shown to have modulatory effects on new neuron production and are themselves candidates for future inclusion in MANGO.

A goal of this resource is to help researchers to identify the cell subtypes and processes regulated in an experiment by applying the ontology to gene set enrichment analyses. The results in **Figure 5** show that this is already possible even with this early version of MANGO. While the example analysis can only demonstrate significant enrichment at a high level in the ontology–as more genes are annotated to the lower-level terms it should become possible to identify focused terms associated with a query gene list. This would be an immense aid to interpretation of high-throughput data such as from microarray experiments, where currently available tools cannot provide the level of detail required to be truly relevant to the field.

We are confident that the number of genes that can be annotated to MANGO will grow steadily if the trend in the literature for a steep increase in adult neurogenesis publications continues as it has over the last two decades.

The provision of a common framework to describe adult hippocampal neurogenesis will hopefully also allow researchers in this field to report gene effects in a standardized way to increase comparability with the rest of the community. By specifically addressing gene effects with respect to terms in the ontology, and explicitly referring to these terms in publications, researchers will be able to directly compare results and ensure that their work optimally integrates into the MANGO knowledgebase.

The present version of MANGO was designed to accommodate some specific needs that arose in the context of research on adult hippocampal neurogenesis that could not be met with the tools offered by existing ontologies and annotation projects. The future extension of MANGO to adult olfactory bulb neurogenesis is envisioned and desired, but a number of substantial hurdles still need to be overcome. In addition, if GO continues to become the standard reference framework, a seamless link between an ontology like MANGO and GO will also be required. Such extensions of the project will be community efforts that must involve also other fields with similar needs. Until then, the database and website will help researchers to quickly gain information about published gene effects on adult neurogenesis. This, too, is no final solution but meant as an invitation to start using the database and develop better questions and a clearer picture of the demands to be put on a resource like MANGO.

## Methods

### Ontology Development

The core ontology was conceived to describe the different states (often referred to as ‘cell types’ in this manuscript) an adult hippocampal stem cell passes through as it becomes a new-born neuron. Based on the distinction of ‘cell types’ described in [8] which has become a standard in the field, the terms were organized in such a way that each term could be considered a sub-type of its parent (the term one step above it in the hierarchy). Terms were linked with “is_a” relationships [24], as appropriate for nested cell-type descriptions (in contrast to “part_of” for anatomical relationships). Thus, for example, every ‘late progenitor’ is a ‘progenitor’, whereas the parent term is broader than its child such that not every ‘progenitor’ is a ‘late progenitor’ (it may be an ‘undetermined progenitor’).

### Definitions

#### Cell types and the core ontology

Stages in the model relate to ‘cell types’, representing the precursor cells and their progeny throughout development (**Fig. 1B**). The populations of cells in the adult subgranular zone are not independent of each other but represent developmental stages. We distinguish:

Type-1: the putative **stem cell** of the adult SGZ with radial glia-like and astrocytic features, including the expression of radial glial marker proteins and GFAP. Their original definition was based on the expression of GFP under the neural enhancer element of the Nestin gene, low proliferative activity, and their radial morphology [25]. It is assumed that a related, even more quiescent long-term stem cell with the same morphology exists [15], [26]–[31], which would be dubbed type-0 or **vertical astrocyte**.

Type-2: the highly proliferative intermediate progenitor cell of the SGZ. We distinguish type-2a (**undetermined progenitor**), that has glial features but lacks the radial morphology of type-1, from type-2b (**determined progenitor**) that shows the first signs of neuronal differentiation [27]. The original distinction was based on the expression of doublecortin (DCX) [25].

Type-3: the lineage determined **neuroblast-like cell** with a more rounded nucleus, first signs of neurite extension and low proliferative activity. This cell type lacks the immature precursor cell markers such as Sox2 or nestin.

The **immature neuron**, after leaving the cell cycle, expresses neuronal marker NeuN and transiently the calcium-binding protein calretinin [32]. Calretinin is initially overlapping with DCX to define a distinct stage (**early immature neuron**). The **late immature neuron** retains calretinin expression without DCX. Calretinin is later exchanged for calbindin, which is also expressed in the granule cells. After this period, the new-born neuron is considered a **mature neuron** and an established **granule cell neuron**. These are distinguished by characteristic mature electrophysiological properties [33]–[35]. At present it is not fully clear, whether calbinin expression and acquisition of the mature electrophysiological phenotype indeed completely coincide. The total number of granule cell neurons is not only dependent on adult but also on embryonic and early postnatal neurogenesis, processes which fall outside the scope of the core ontology. Some studies have reported gene effects on mature granule cell neurons, and gene expression in these cells, and we have also included this information in the database, because new neurons are part of the population of granule cells.

The root term of the core ontology is **adult hippocampal neurogenic cell** which encompasses the proliferating **precursor** cell type and the post mitotic **new neuron** lineage. Other lineages which do not result in the generation of new neurons are not included in this core ontology as their relationship to the stem cell and other cell types has not yet been absolutely established. The term precursor is understood to include both the self-renewing stem cells (type-0 and type-1) as well as the **progenitor** pool which aside from the undetermined type-2a stage, also includes the lineage-determined **late progenitor** cell types (type-2b and type-3).

#### Other markers

Despite their being outside the core ontology, we have also annotated information for the new-born **astrocyte** in the hippocampal dentate gyrus as well as the extremely rare adult-born **oligodendrocyte**. For the latter the lineage relationship in vivo is unclear. For astrocytes the distinction between postmitotic astrocytes (‘horizontal astrocytes’ [26]) and the stem cells with astrocyte-like properties must be made. Horizontal astrocytes in mice express S100b, whereas the stem cells do not [27]. Commonly, ‘astrogenesis’ requires the detection of S100b. The maintenance of stem cells in some sense also represents astrogenesis but should clearly be distinguished.

Although cell types are largely defined based on expression of marker proteins, a key problem is that several widely used markers are equivocal with respect to the cell type identified. While multiple markers are required to properly define the cell type, these are often used alone as a surrogate for detailed phenotyping. This is the situation, for example, with DCX, a very commonly used marker, expressed in both progenitor cells (type-2b or type-3) and immature neurons. In such cases, DCX staining is not able to accurately place a cell into a discrete ontological category. The markers DCX, calretinin and calbindin are so commonly used in the field in this way, that the annotation tool (and hence the ontology) must do justice to this usage, even if it is problematic. A special case of this type of common usage involves the measurement of proliferation, which is almost always carried out using the thymidine analog bromodeoxyuridine (BrdU) which incorporates into the DNA of dividing cells during S-phase. In many studies new neurons are only identified by NeuN expression in cells birthdated with BrdU at an earlier time point. In most cases their quantification represents ‘numbers’ of ‘new neurons’–a key outcome measure, and end point of most of studies on adult neurogenesis. This measure is dependent, however, on the labeling interval chosen. Three to four weeks after cell cycle exit is regarded as a safe margin [36]. BrdU is also often used to estimate the proliferation of precursor cells, but as it indiscriminately labels all dividing cells, detailed cell type information is obtained. The relationship between BrdU labeling and the quiescent type-0 cell is difficult to determine–there is not yet an established method to distinguish type-0 cells histologically and only a small (and unknown) fraction of these cells will be labeled in any experiment. BrdU can also label some other non-neurogenic cell types in the hippocampus, preventing it being a perfect marker of precursor cells.

#### Processes

Processes in adult neurogenesis are the actions by the different cell types at the different stages of development.

**Proliferation** refers to the mitotic activity of precursor cells in the SGZ which, for example, can be measured with (1) a pulse of BrdU (one or more doses within hours) and consecutive detection of BrdU not later than 24 h after the first dose, or (2) the detection of endogenous cell cycle-associated markers such as mKi67, PCNA, Mcm2, pH3. There are limitations; depending on the post-injection interval (in the BrdU method) or the antigen type, numbers might detect cells in discrete cell cycle phases (BrdU: S-phase; Ki67: all except G0 and early G1; pH3: M-phase; etc.), and BrdU dosage influences estimates. Within-experiment comparisons between groups are less affected by these issues than between-experiment comparisons.

The distinction between reduced cell proliferation and reduced precursor cell survival is not addressed in most studies, and is generally inaccessible using constitutive mutants due to loss of gene function throughout brain development. ‘Proliferation’ thus stands for ‘number of presently proliferating cells’ irrespective of the underlying cause. Ideally, a ‘proliferation’ phenotype would be standardized to the available number of precursor cells that *could* maximally proliferate, i.e. the proliferative fraction [37], [38].

Conceptually, from the perspective of stem cell biology, ‘proliferation’ also stands for two different aspects of precursor cell behavior: self-renewal and expansion. Self-renewal maintains the population of precursor cells, whereas expansion increases the number of cells that might differentiate. Most experimental assays are (still) blind to this distinction. But increased baseline proliferation (e.g. in a mutant) might stand for ‘increased self-renewal’, whereas increased proliferation after some acute stimulus does not.

The **differentiation** phenotype requires that, among the surviving newborn cells, an altered proportion of cells become neurons. To qualify as a separate phenotype, differentiation must be independently affected and not be merely a result of increased survival across all cell types (neurons and glia). The term ‘differentiation’ as it is used in the adult neurogenesis literature is also problematic, because it remains unclear if the effect is a consequence of an altered fate choice decision or of a selection effect. A differentiation phenotype stands for an increased proportion of new neurons among the number of surviving newborn cells in the adult hippocampus or the increased number of neuronal-determined precursor cells, as assessed, for example, by the expression of *Dcx*, *Neurod1* or *Prox1*.

**Migration** refers to the translocation of a newborn cell from the place of proliferation to the place of terminal maturation and integration and has so far been measured in only very few studies. Migration is actually very limited in adult hippocampal neurogenesis [36]. From what is known, migration occurs at the level of progenitor cells, mostly type-3 and shortly after cell cycle exit. It is associated with the expression of DCX and PSA-NCAM. Although an initial contact to the radial glia-like precursor cells might be maintained for some time [39], migration, however little, generally must occur [40]. In pathological situations ectopic or overshooting migration can occur.

We define **survival** as the continued existence of a cell in the course of development. This is more than just the consequence of ‘negative regulation of apoptosis’, because survival can be positively influenced and controlled.

In the literature, quantification of ‘survival’ requires the BrdU method and assessment at one early and one late time point after BrdU labeling. The intervals can vary; in most publications, survival relates to an interval of 3–4 weeks after BrdU injection. To qualify as a quantifiable phenotype, ‘survival’ has to represent the survival rate; the ratio of cell numbers at the late versus the early time point–a value not directly assessed in most studies.

Generally, the survival rate of newborn cells cannot be assessed if proliferation has not been measured in the same experiment. Increased proliferation results in increased numbers of ‘surviving’ cells without an independent effect on survival rate, merely as a result of the increased baseline. Survival measurements are confounded by effects on proliferation.

Most often reported is the number of new neurons, which often infers altered survival, although without making the above distinctions.

Few publications to date have dealt with specifics of dendrite development (**dendritogenesis**) in the adult dentate gyrus. In adult hippocampal neurogenesis, dendrite development appears to occur during the phase of DCX expression, beginning with type-2b/3 progenitor cells but mostly occurring postmitotically [40], [41].

As yet, extremely little is known about axon development (**axonogenesis)** during adult neurogenesis. We know that it takes place early after exit from the cell cycle. In mice and rats the axon has reached the target zone in CA3 within approximately 10 days [42]–[44]. This implies that axon development is associated with the late, calretinin, phase of neurogenesis although no causal relationship between calretinin expression and axonal development is yet known. The establishment of connectivity in CA3 with the formation of synapses in the characteristic boutons of the mossy fibers [45] is part of axonal development.

**Maturation** is defined here as the qualitative events besides dendrite and axon elongation that occur between the initiation of neuronal differentiation and full functional network integration, including the expression of characteristic ion channels, specifics of synapse formation, etc. The term is not consistently used in the literature: sometimes maturation stands simply for net neurogenesis or even ‘differentiation.’

#### Outcomes

Information in the literature is usually not structured as described above, but is related to a number of surrogate outcome measures–the most relevant being active granule cell function and cell numbers. In some sense these are the end points of neurogenesis.

Underlying this is the usually implicit assumption that the ultimate outcome of adult hippocampal neurogenesis is a change in network architecture leading to altered hippocampal function. We thus distinguish the actually measured end point from the ‘process’ that leads to this outcome.

Defined here as the functional integration of the new neuron into the network, active granule cell **function** arguably represents the end point of development and is thus an outcome measure. Nevertheless, immature neurons serve a distinct and different function than the mature fully integrated neurons such that the transient immature function could be seen as preparing the way for the development of the lasting mature function.

Adult neurogenesis leads to changes in cell **numbers**, be it new neurons or precursor cells. Most studies address changes in cohorts of cells, usually identified by labeling with BrdU. These cohorts represent subsets of the target population, many of which were not labeled because they were not in S-phase at the time of BrdU injection. Net changes in entire populations have been addressed in only few studies. An increasing number of studies use total DCX-positive cell number as a surrogate measure of adult neurogenesis. Because DCX expression spans from proliferative precursor cells to postmitotic new neurons, this measure is influenced by many, usually unknown, variables. While obviously an interesting number in direct comparisons, it is unclear exactly how useful it is as an absolute measure.

In many cases, the literature contains only information about gene **expression** in a particular cell type in the context of adult neurogenesis without further assessment of that gene’s role in that cell type and hence its ultimate contribution to adult neurogenesis. Although gene expression is biologically no end point in itself, an expressed gene is an outcome and can be representative of a process.

### Gene List Curation

Query of the GoGene database (http://www.gopubmed.com) [46] was made on April 19, 2011. The search term used was [adult AND neurogenesis]. The 1735 hits retrieved were filtered for the MeSH term “Hippocampus” (ID: D006624; http://www.nlm.nih.gov/mesh/).

Ongoing update of the database is provided by an automated search: “((“adult neurogenesis”) OR ((adult OR postnatal) AND (“stem” OR “progenitor*” OR “precursor*” OR “new born”))) AND (hippocamp* OR “dentate gyrus” OR sgz OR “subgranular”) AND (gene OR transcript* OR protein OR rna OR expression OR receptor OR agonist OR antagonist OR transgen* OR knock*)”. Curation of new manuscripts is carried out manually, and the updated database is made publicly available from the website (http://adult-neurogenesis.de/resources/mango) as discrete releases to ensure replicability of analyses. The current release at the time of writing is 2.0.

### Analyses

Functional annotation was done using the ‘Functional Annotation Clustering’ tool from the DAVID project (http://david.abcc.ncifcrf.gov/). The protein interaction network was generated by submitting gene symbols to STRING (http://string-db.org/) [47] and adding the top 10 interaction partners from the STRING database. Only interactions with a STRING confidence score of 0.4 and above were used. For enrichment analysis, the traits ‘PROL’ and ‘NEUR’ (Record IDs 10795 and 10797 from the GeneNetwork database; http://genenetwork.org) were correlated to gene expression data from whole brain (UTHSC Brain mRNA U74Av2 (Aug05) RMA) using a threshold of an absolute Pearson correlation of 0.4 or higher to ‘PROL’ or ‘NEUR’–but not the ‘PROL’ × ‘NEUR’ intersection. Enrichment for MANGO terms was calculated using the hypergeometric test as implemented by the *phyper* and *dhyper* functions in R (http://cran.r-project.org/) and the resulting *p*-values were corrected for multiple testing using the Bonferroni method. For enrichment testing, the *Mus musculus* background gene list from the DAVID project was used.

All databases were queried on 8th February 2012 to generate the figures shown.

### Database

The MANGO website is a Java-based web application running on Apache Tomcat server with MySQL database back-end. The software was developed with SpringSource Tool Suite™ and employs Grails as a web application framework. Web pages were tested for proper content rendering in all major browsers.

The website is freely available and does not require any registration. Results of all search queries can be downloaded as tab-delimited text files.

## Supporting Information

Table S1 **The complete MANGO database.** Each row of the table contains all reported effects of a single gene in a single manuscript. Genes annoated to the MANGO database are listed as official MGI mouse gene symbol, official gene name and common names, and NCBI GeneID. Abbreviations for cell types/markers are: Ast, astrocytes; BrdU, cells positive after labeling with 5-bromodeoxyuridine; Cb, calbindin-expressing cells; Cr, calretinin-expressing cells; Dcx, doublecortin-expressing cells; Gc, mature granule cells; In, immature neurons; Nn, total new-born neurons; Oli, oligodendrocytes; Pc, precursor cells; T0, type-0 cells; T1, type-1 cells; T2, total type-2 cells; T2a, type-2a cells; T2b, type-2b cells; T3, type-3 cells. Detailed cell type descriptions are given in the ‘Methods’ section of the accompanying manuscript. The processes comprising the columns of the table are annotated with the cell types affected as well as the direction of the effect: +, positive; −, negative; ±, no change. Studies only dealing with gene expression are marked with one asterisk, *, studies involving the extrinsic manipulation of that gene, for example by pharmacological intervention, two asterisks, **, and studies dealing with the direct manipulation of the gene, e.g. a transgenic or knockout experiment, three asterisks, ***. Codes describing the species in which the reported work was done are: m, mouse; r, rat; mon, monkey; gp, guinea pig.
(XLS)

Table S2 **Genes used for enrichment analysis.** Gene lists were obtained by correlating data for two adult hippocampal neurogenesis phenotypes with brain transcript expression values measured in the same strains of a genetic reference population. For more details on this experiment please refer to the original publication [18]. Raw data are available at GeneNetwork (http://genenetwork.org). The table consists of three spreadsheets; **PROL** and **NEUR** contain columns with the gene list returned from the correlation calculation, discontinued gene symbols, and the current Entrez GeneID and Gene Symbol for each gene used in the analysis. In addition, output from the hypergeometric tests, including the *p*-values adjusted for multiple testing using the Bonferroni method, and the query genes identified in each MANGO term.
(XLS)
